# Randomized Clinical Trial to Evaluate the Morphological Changes in the Adventitial Vasa Vasorum Density and Biological Markers of Endothelial Dysfunction in Subjects with Moderate Obesity Undergoing a Very Low-Calorie Ketogenic Diet

**DOI:** 10.3390/nu14010033

**Published:** 2021-12-23

**Authors:** Enric Sánchez, Maria-Dolores Santos, Maitane Nuñez-Garcia, Marta Bueno, Ignacio Sajoux, Andree Yeramian, Albert Lecube

**Affiliations:** 1 Obesity, Diabetes and Metabolism (ODIM) Research Group, IRBLleida, University of Lleida, 25198 Lleida, Spain; esanchez@irblleida.cat (E.S.); lolasantrey@hotmail.com (M.-D.S.); mbueno.lleida.ics@gencat.cat (M.B.); 2 Endocrinology and Nutrition Department, Arnau de Vilanova University Hospital, 25198 Lleida, Spain; andreeyeramian85@gmail.com; 3 Pronokal Group, 08009 Barcelona, Spain; Maitane.Nunez@pronokalgroup.com (M.N.-G.); Ignacio.S@pronokal.com (I.S.); 4 Centro de Investigación Biomédica en Red de Diabetes y Enfermedades Metabólicas Asociadas (CIBERDEM), Instituto de Salud Carlos III (ISCIII), 28029 Madrid, Spain

**Keywords:** vasa vasorum, weight loss, very low calorie ketogenic diet, obesity, multidisciplinary approach, pronokal method

## Abstract

Weight loss after bariatric surgery decreases the earlier expansion of the adventitial vasa vasorum (VV), a biomarker of early atheromatous disease. However, no data are available regarding weight loss achieved by very low calorie ketogenic diets (VLCKD) on VV and lipid-based atherogenic indices. A randomized clinical trial was performed to examine changes in adventitial VV density in 20 patients with moderate obesity who underwent a 6-month very low calorie ketogenic diet (VLCKD, 600–800 kcal/day), and 10 participants with hypocaloric diet based on the Mediterranean Diet (MedDiet, estimated reduction of 500 kcal on the usual intake). Contrast-enhanced carotid ultrasound was used to assess the VV. Body composition analysis was also used. The atherogenic index of plasma (log (triglycerides to high-density lipoprotein cholesterol ratio)) and the triglyceride-glucose index were calculated. Serum concentrations of soluble intercellular adhesion molecule 1 (sICAM-1), and soluble vascular cell adhesion molecule 1 (sVCAM-1) were measured. The impact of weight on quality of life-lite (IWQOL-Lite) questionnaire was administered. Participants of intervention groups displayed a similar VV values. Significant improvements of BMI (−5.3 [−6.9 to −3.6] kg/m^2^, *p* < 0.001), total body fat (−7.0 [−10.7 to −3.3] %, *p* = 0.003), and IWQOL-Lite score (−41.4 [−75.2 to −7.6], *p* = 0.027) were observed in VLCKD group in comparison with MedDiet group. Although after a 6-months follow-up period VV density (mean, right and left sides) did not change significantly in any group, participants in the VLCKD exhibited a significantly decrease both in their atherogenic index of plasma and serum concentration of sICAM-1. A 6-month intervention with VLCKD do not impact in the density of the adventitial VV in subjects with moderate obesity, but induces significant changes in markers of endothelial dysfunction and CV risk.

## 1. Introduction

The prevalence of obesity is increasing worldwide [1]. In the United States, 34.9% of adults have obesity [2]. In Spain, the prevalence reaches 21.6%, and it is more common in women and with increasing age [3]. Obesity is one of the world’s deadliest diseases, with more than 2.8 million deaths annually, and it has a marked impact on cardiovascular (CV) morbidity and mortality [4,5,6]. It has been reported that CV disease tends to occur at an earlier age in patients with obesity [7]. Therefore, the early diagnosis of asymptomatic atherosclerosis may be key to preventing CV events and reducing mortality in persons with obesity. 

The vasa vasorum (VV) form a microvascular network situated in the adventitial layer of medium and large arteries. Their function is to supply nutrients and oxygen to the cells of the vessel wall [8]. Proliferation of the VV is the earliest defensive response to endothelial damage by potential stressors, such as hypoxia, inflammation, and hyperglycemia [9,10,11]. There is an association between VV proliferation and inflammation, intraplaque hemorrhage, and thin-cap fibroatheromas [12]. Thus, patients with morbid obesity show an increased VV density compared with subjects with a normal weight and overweight [13]. Our group has demonstrated how weight loss after bariatric surgery has a favorable impact on the carotid wall, reducing VV density [13]. However, today there is still no information on changes in VV density after weight loss through lifestyle modification. 

Although ketogenic diets restrict carbohydrate intake, the specific daily calorie intake, macronutrient composition and duration vary between each intervention [14]. Very low calorie ketogenic diets (VLCKD) are characterized by a low carbohydrate content (<50 g/day), 1–1.5 g of protein/kg ideal body weight, less than 10 g of fat/day, and a daily intake of between 500 and 800 kcal. VLCKDs have been proposed as an effective weight loss method for patients with obesity [15,16]. Although this nutritional intervention is widely accepted, there are some potential negative cardiovascular effects still under discussion [17]. No previous research has been done on the effect of VLCKDs on VV. To study this relationship and further investigate VV dynamics, we designed a randomized clinical trial to evaluate the morphological changes of the carotid arterial wall in subjects with moderate obesity who underwent weight loss treatment with a commercial VLCKD weight-loss program (PNK Method^®^).

## 2. Materials and Methods

### 2.1. Ethics Approval

The protocol was accepted by the University Hospital Arnau de Vilanova ethics committee (CEIC-1966L) and previously registered in the ClinicalTrials.gov (Identifier: NCT01865448; last access date: 19 December 2021). The clinical trial was carried out in accordance with the ethical guidelines of the Helsinki Declaration and Spanish legislation regarding the protection of personal information. Written informed consent was obtained from all participants before their entry into the study. Reporting has followed the CONSORT guideline for randomized trials [18].

### 2.2. Study Design and Participants

Randomized, controlled, single-center clinical trial that analyzed changes in adventitial VV density and in the biological markers of endothelial dysfunction in patients with moderate obesity undergoing a multidisciplinary, structured program for weight loss using a VLCKD over a 6-month period. The study was performed in the Arnau de Vilanova University Hospital in Lleida, Spain. This hospital is a Center of Obesity Management accredited by the European Association for the Study of Obesity. The study was open for recruitment from May 2019 to December 2019, when the target sample size was reached. 

**Figure 1:**
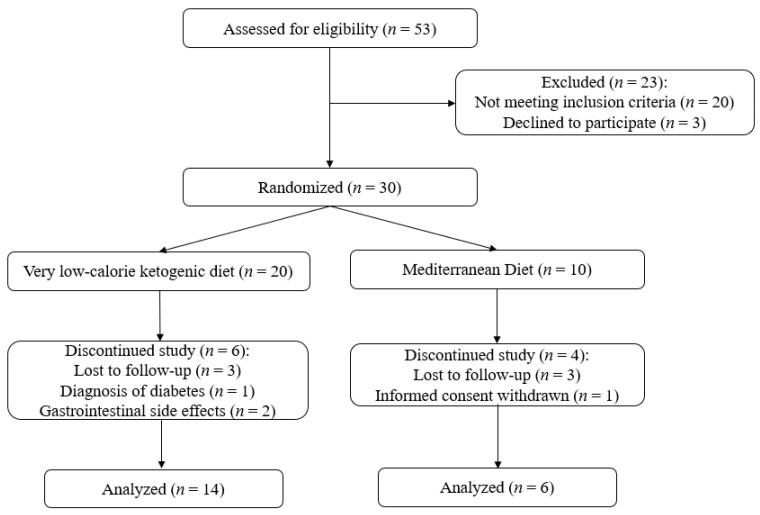
CONSORT 2010 flow diagram for the study.

The study flow chart is displayed in Figure 1. Eligible participants included men and women aged 18–65 years, with a body mass index (BMI) between 35.0 and 39.9 kg/m^2^. All patients provided written consent confirming their willingness to participate in the study and had to agree to participate in a designed weight loss program. The following exclusion criteria were applied: (i) previous bariatric surgical procedure; (ii) any type of diabetes mellitus at any phase of the study (using the American Diabetes Association criteria); (iii) drug addiction or psychological disturbances including eating disorders or alcoholism; (iv) chronic kidney disease (estimated glomerular filtration rate <60 mL/min/1.73 m^2^) or liver cirrhosis; (v) history of CV or cerebrovascular disease; (vi) pregnant or breast-feeding women; and (vii) individuals with a known allergy to sulfur hexafluoride ultrasound contrast.

### 2.3. Interventions

Using the standard deviation of adventitial VV from a previous study, we determined that the minimum necessary sample size was 30 subjects [19]. Therefore, patients were randomized 2:1 into two groups to receive either a commercially available VLCKD (*n* = 20) or a standard-of-care approach based on the Mediterranean diet (MedDiet, *n* = 10) for weight loss purposes. Random allocation was done by an external company using a computer-generated randomization list. All individuals underwent clinical assessment by a specialist endocrinologist and dietitian each month throughout the course of the study. Aiming for congruency between both groups, adequate dietary habits, and intense physical activity indications (140 to 280 min weekly) were provided by expert support staff. 

The VLCKD group completed a diet according to the PNK^®^ Method, a commercial weight loss program based on a high biological value protein preparation [20]. Each protein preparation contained 15 g protein, 4 g carbohydrates, 3 g fat, and provided 90–100 kcal. The VLCKD arm was supplemented with 500 mg/day docosahexaenoic acid (DHA) [20]. During the firsts three months of the treatment period patients were kept in ketosis (“active stage”) of the method. This stage consists of a very low carbohydrate (<50 g daily from vegetables with a low glycemic index), very low calorie (600–800 kcal/day), and very low fat (only 10 g of extra virgin olive oil per day) diet. The quantity of high-biological-value proteins was between 0.8 and 1.2 g per kilogram ideal body weight, to cover minimum body requirements and to avoid the loss of fat-free mass. Supplements of vitamins and minerals, including potassium, sodium, magnesium, and calcium, and DHA (docosahexaenoic acid) were provided according to international recommendations [21]. This active stage was followed by a 2-month VLCKD in which one of the protein servings was substituted with a natural protein (e.g., meat or fish). Finally, a 1-month controlled diet in which a second serving of low-fat natural protein was substituted for the second serving of biological protein preparation. These timings were personalized for each patient. 

Participants allocated to the standard hypocaloric diet (estimated reduction of 500 kcal on the usual intake) based on the Mediterranean diet (MedDiet) followed the same monthly visits as the VLCKD group. MedDiet consisted of 50% carbohydrates (including whole grains and vegetables), approximately 15% protein, and 35% fat (including extra virgin olive oil and nuts).

Finally, 20 subjects with normal weight or overweight (BMI 24.2 ± 2.0 kg/m^2^) were used as a baseline control group only for the evaluation of the adventitial VV density. Those individuals were matched for sex and age (±2 years) with the global interventional group. 

### 2.4. Contrast-Enhanced Carotid Ultrasound

Contrast-enhanced carotid ultrasound was performed using the Siemens Sequoia 512 ultrasound system, equipped with a 15L8W linear array probe and with ultrasound contrast software (Cadence contrast Pulse Sequencing technology). A phospholipidic shell containing sulfur hexafluoride (Sonovue, Bracco Spa, Milan, Italy) was used as the contrast agent. Full technical details have been described previously [13,22]. Results are displayed for the right and left sides, and the mean VV signal was calculated for the 2 sides. All ultrasound studies were stored digitally for retrospective analysis and were quantified by a blinded investigator. Baseline evaluation was followed by a repeat evaluation 6 months after the nutritional intervention.

### 2.5. Body Composition, Anthropometric Data, and Quality-of-Life Evaluation

Body composition at baseline and after 6 months of follow-up was analyzed using a segmental body composition device (Tanita MC-580, Amsterdam, The Netherlands). Weight and height were estimated in light clothing and without shoes using standard equipment, to the nearest 0.5 kg and 1.0 cm, respectively. BMI was defined as the body weight (kg) divided by the square of the body height (m). Waist circumference was measured with the participant in a standing position, using a non-elastic tape with an accuracy of 0.1 cm places in the horizontal plane between the iliac crest and the lowest rib. To avoid inter-observer and inter-device variability, all measurements were taken by a single experienced investigator using the same devices.

The Impact of Weight on Quality of Life-Lite (IWQOL-Lite) was administered to quantitatively assess the individual’s perception of how weight affected their day-to-day life [23]. This questionnaire comprises 31 items grouped into 5 dimensions: physical functioning, self-esteem, sexual life, public distress, and work. The measure provides scores for each dimension and a total score.

### 2.6. Laboratory Assessment

Blood samples were taken by direct puncture of the antecubital vein after an overnight fast of 8 h and just before administration of the contrast agent. Blood samples were separated by centrifugation (2.000× *g* at 4 °C for 20 min) and analyzed in the clinical laboratory of our hospital using standard methods. The atherogenic index of plasma (AIP), which is considered a significant predictor of CV risk, was calculated as the logarithmically transformed ratio of molar concentrations of triglycerides to HDL-cholesterol [24]. The triglyceride-glucose index, a novel biomarker that has been associated with morphological characteristics of the atheroma plaque and CV events, was also measured [25]. Finally, serum concentrations of soluble intercellular adhesion molecule 1 (sICAM-1) and soluble vascular cell adhesion molecule 1 (sVCAM-1) were measured on serum samples. All measurements were made in duplicate using an enzyme-linked immunosorbent assay (ELISA) kit, following the instructions of manufacturer (Abcam, Cambridge, UK).

### 2.7. Statistical Analysis

A normal distribution of the variables was established using the Kolmogorov–Smirnov test, and data were expressed as the mean ± SD or as percentages. The main clinical data across the three groups were compared using Student’s *t*-test or the ANOVA test for continuous variables. Pearson’s chi-squared test was used for categorical data. In addition, the relationship between continuous variables was assessed using the Pearson correlation test. Changes in the biomarkers and other parameters were evaluated using a paired *t*-test. For this exploratory pilot study to prove a concept, we did not perform a power analysis to estimate sample sizes.

All *p* values were based on a 2-sided test for statistical significance. Significance was accepted as a *p* value less than 0.05. Statistical analyses were performed using the SPSS software (IBM SPSS Statistics for Windows, Version 27, Armonk, NY, USA).

## 3. Results

At baseline, no significant differences were observed between the intervention groups (VLCKD and MedDiet) regarding age, sex, anthropometry, clinical and metabolic parameters, and quality of life (Table 1). 

**Figure 2:**
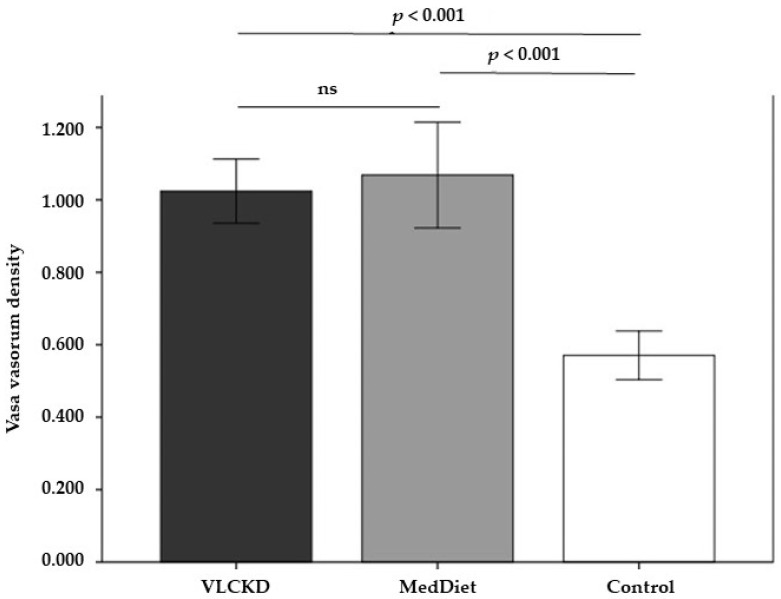
Adventitial vasa-vasorum density at baseline in the 3 groups (mean and 95% CI). Control group comprised 20 subjects with normal weight or overweight (BMI 24.2 ± 2.0 kg/m^2^) matched by sex and age to the participants with moderate obesity.

Although adventitial VV density was significantly lower in subjects with normal weight or overweight in comparison with participants with moderate obesity, no differences were observed (mean, left and right sides) between the VLCKD and MedDiet groups at baseline (Table 1, Figure 2). Similarly, at baseline, no intergroup differences were observed in the biological markers of endothelial dysfunction (Table 1).

**Figure 3:**
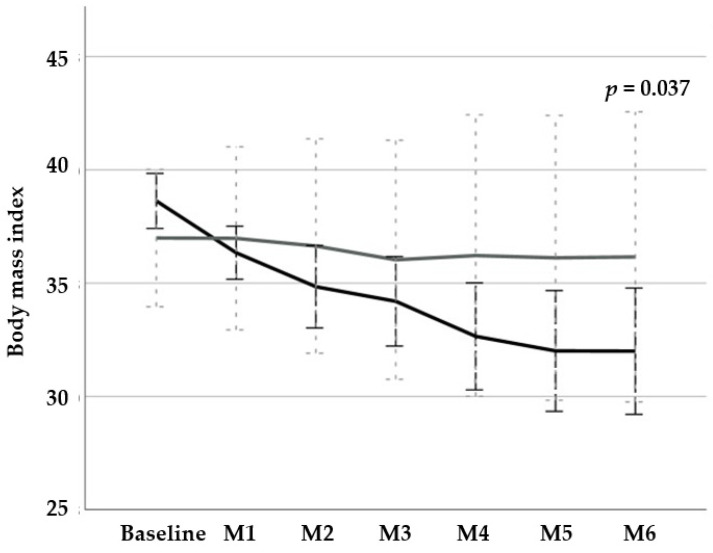
Plot displaying dynamics of body mass index values at baseline and during the 6-month follow-up period in the study population. Black line: very low calorie ketogenic diet; grey line: Mediterranean diet; M: month.

After a follow-up period of 6 months, participants allocated to the VLCKD group, in comparison with the MedDiet group, experienced a significant decrease in their BMI (−5.2 kg/m^2^ [95% confidence interval (CI) −8.3 to −2.0 kg/m^2^], *p* = 0.003) and total body fat (−5.7% [95% CI −10.4% to −1.0%], *p* = 0.022), without differences in lean body mass (−5.2% [95% CI 7.0% to −17.4%], *p* = 0.370), and a significant improvement in their quality of life (−31.7 [95% CI −66.7 to 3.4], *p* = 0.041) (Table 2, Figure 3). Participants in the VLCKD group experienced improvements in physical functioning (−21.3 [95% CI −34.7 to −7.9], *p* = 0.009), self-esteem (−14.3 [95% CI −20.9 to −7.7], *p* = 0.003), sexual life (−4.8 (95% CI −8.3 to 1.2), *p* = 0.020], and public distress [−6.4 [95% CI −12.2 to −0.6], *p* = 0.037). Only the work dimension remained unaffected.

**Figure 4:**
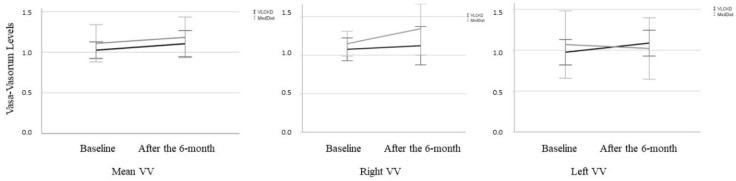
Plot displaying dynamics of vasa-vasorum results at baseline and after the 6-month follow-up period in the intervention groups.

No differences were detected in the mean adventitial VV density between the 2 intervention groups at the end of the follow-up period (Table 3, Figure 4). Right and left VV densities were also similar in the 2 groups after the follow-up period (Table 3). However, at the end of the follow-up period, participants in the VLCKD group showed a significant fall in the serum concentration of ICAM-1 in comparison with the MedDiet group (−60.9 [95% CI −115.0 to −6.8] ng/mL, *p* = 0.029). In addition, there was a significant decrease in the AIP between baseline and the end of the study in the VLCKD group (Table 4). sVCAM-1 showed no treatment effect in either group.

Finally, two patients in the VLCKD group reported adverse events, mainly affecting gastrointestinal system. Both patients were finally excluded from the study. Furthermore, one patient in the MedDiet group reported a serious adverse event. This event was a non-fatal episode of heart failure that required hospitalization for 3 days, and which resolved after treatment with beta-blockers.

## 4. Discussion 

The link between obesity and CV diseases is well recognized and is predominantly related to increased adiposity. However, the pathophysiological mechanisms have not been fully elucidated. This study was therefore designed to investigate the morphological changes in the carotid arterial wall induced by different weight-loss interventions in subjects with moderate obesity. Our data support the hypothesis that a short period of 6 months is insufficient to induce morphological changes in the carotid wall, but that such interventions can produce improvements in the serological markers of endothelial dysfunction, such as ICAM-1, and in the lipid-based atherogenic indices. These positive findings may be related to a 5-point reduction in the BMI and an almost 6% reduction in total body fat. The VLCKD, as a part of a multicomponent strategy and under strict medical supervision, is an effective plan for the treatment of obesity [15,16] and type 2 diabetes [26,27]. Although some reports suggest that ketogenic diets have been associated with higher CV risk [17], our data do not support this hypothesis and show a positive safety profile with this weight loss approach.

The VV nourish the wall of medium and large arteries [28]. However, their proliferation is seen in the initial stages of atherosclerosis. In pigs fed with a high-fat diet, VV proliferation was detected before the development of intimal thickening and even before the onset of endothelial dysfunction [29]. This discovery suggests that factors other than intimal hypoxia, such as a dysfunctional perivascular adipose tissue, may promote VV proliferation in the atheromatous process [30,31]. In obesity, adipocytokines from the perivascular adipose tissue promote adventitial inflammation, neovascularization, and neointima formation, playing a key role in the pathogenesis of atheromatous changes; the VV may transport inflammatory cells between the neointima and the perivascular adipose tissue [32]. Previous research by our group showed that neovascularization, measured by adventitial VV density, was higher in patients with morbid obesity than in normal weight subjects [13]. This increase in VV density was especially high among subjects with sleep apnea-hypopnea syndrome, reinforcing the role of nocturnal hypoxia in the genesis of atheromatous disease [33]. It has been also shown that adventitial VV density decreases significantly 6 and 12 months after bariatric surgery [13,22]. However, the present study found no effect on VV density after a 6-month weight-loss intervention with a VLCKD or MedDiet. This lack of effect may be due in part to the short follow-up period, the lower baseline BMI, and the smaller reduction in BMI than occurred in the studies with bariatric surgery [13,22].

VLCKD has been proposed as an effective weight loss method for patients with obesity [15,16], including its use in the preoperative setting for bariatric surgery [34]; it has even been included in the preoperative consensus statement from the Italian Society of Endocrinology [27]. Our results agree with previous studies comparing the VLCKD with a hypocaloric diet based on the MedDiet, showing VLCKD to be associated with marked reductions not only in BMI, but also in total body fat and waist circumference, all of which are related to the incidence of CV disease [35,36,37]. It has been suggested that nutritional ketosis and DHA supplementation may be added to the greater weight loss as responsible for these beneficial effects [15,20,38]. Dietary carbohydrate restriction increases the production of the ketone body ß-hydroxybutyrate, shifting tissue cross-talk from a proinflammatory to an anti-atherogenic environment, addressing the residual inflammatory risk and reducing most of the atherosclerotic CV disease biomarkers [39]. The nutraceutical supplementation of VLCKD with commercially available DHA produces both vaso- and cardioprotective responses that have been widely demonstrated both in vitro and in vivo [40]. In addition, VLCKDs have also been associated with reductions in visceral adiposity, at a rate of 505 g/week [41]. It must be highlighted that the loss of weight and fat mass in our study were achieved without changes in fat-free mass, avoiding the risk of sarcopenia-related physical disability, frailty, poor quality of life, and mortality [42,43,44]. In point of fact, a significant improvement in quality of life was reported by participants in the VLCKD group in our study. Similar data have previously been reported in the physical function and self-esteem scores [45]. We also observed improvements in sexual life and public distress.

Finally, we observed a marked fall in the serum concentration of sICAM-1, though not of sVCAM-1, in the VLCKD group in our study, as well as a significant decrease in the AIP. These effects are consistent with the findings in other studies [46]. sICAM-1 appears to be an earlier marker of endothelial injury than sVCAM-1 [47]. This information has been clearly established by prospective studies with several years of follow-up [48,49]. Consequently, we suggest that 6 months of a VLCKD in subjects with moderate obesity and without previous CV disease is sufficient to ameliorate endothelial dysfunction associated with obesity. Further studies are needed to determine whether a longer period of time or greater weight loss could provoke positive changes in the adventitial VV density.

This study has certain limitations. The number of participants in each arm of the study was small for which our conclusions need to be replicated in the future in larger studies. Other relevant information that could influence the results, such as menopausal status, was not available in our study [50]. The gold standards for the assessment of body adiposity are dual-energy X-ray absorptiometry and magnetic resonance imaging [51,52]. However, previous studies have reported similar results using dual-energy X-ray absorptiometry, multifrequency bioelectrical impedance, and air displacement plethysmography [15]. In addition, a longer follow-up period would improve our data. Finally, the two interventions were not compared in an iso-caloric manner.

## 5. Conclusions

In conclusion, a 6-month intervention with a VLCKD did not impact the density of the adventitial VV in subjects with moderate obesity, though it did produce a reduction in markers of endothelial dysfunction and of atheromatous disease. These results are associated with a marked decrease in body weight and total body fat, without affecting fat-free mass, compared with hypocaloric diets based on the MedDiet. VV measurement is complex and time-consuming. We recommend monitoring sICAM-1 levels in subjects with obesity to achieve a better classification of CV risk.

## Figures and Tables

**Table 1:** Baseline characteristics of the participants in the study according to diet.

	VLCKD (*n* = 20)	MedDiet (*n* = 10)	*p* Value
Age (years)	40.7 ± 9.6	39.7 ± 9.0	0.985
Women, *n* (%)	14 (70.0)	8 (80.0)	0.834
BMI (kg/m^2^)	38.1 ± 1.6	37.5 ± 2.5	0.284
WC (cm)	112.4 ± 7.2	111.3 ± 10.1	0.631
TBF (%)	42.4 ± 2.9	42.0 ± 4.1	0.790
FFM (%)	55.0 ± 5.6	56.1 ± 7.5	0.572
SBP (mmHg)	130.1 ± 15.9	134.8 ± 17.3	0.394
FPG (mmol/L)	107.0 ± 38.3	99.5 ± 14.3	0.579
HbA1c (%)	5.6 ± 1.2	5.3 ± 0.3	0.504
Total cholesterol (mg/dL)	192.1 ± 32.2	179.8 ± 30.5	0.356
c-LDL (mg/dL)	120.9 ± 30.6	110.5 ± 23.0	0.387
c-HDL (mg/dL)	47.5 ± 12.7	46.9 ± 14.6	0.922
Triglycerides (mg/dL)	138.7 ± 68.8	144.4 ± 72.4	0.839
AIP	0.5 ± 0.2	0.7 ± 0.2	0.667
TyG index	8.8 ± 0.4	9.1 ± 0.3	0.682
Creatinine (mg/dL)	0.67 ± 0.1	0.67 ± 0.2	0.964
IWQOL-Lite	79.0 ± 27.5	78.3 ± 35.9	0.539
Baseline mean VV	1.02 ± 0.2	1.06 ± 0.2	0.557
Baseline right side VV	1.07 ± 0.2	1.15 ± 0.2	0.770
Baseline left side VV	1.00 ± 0.3	1.06 ± 0.4	0.292
sICAM-1 (ng/mL)	416.4 ± 110.3	385.7 ± 159.3	0.527
sVCAM-1 (ng/mL)	1508.9 ± 201.3	1576.8 ± 321.4	0.499

Data are mean ± SD or *n* (percentage). BMI: body mass index; WC: waist circumference; TBF: total body fat; FFM: fat-free mass; AIP: atherogenic index of plasma; TyG index: triglyceride-glucose index; SBP: systolic blood pressure; FPG: fasting plasma glucose; HbA1c: glycated hemoglobin; c-LDL: low-density lipoproteins cholesterol; c-HDL: high-density lipoproteins cholesterol; IWQOL-Lite: Impact of weight on quality of life-lite; VV: vasa vasorum; sICAM-1: soluble intercellular adhesion molecule 1; sVCAM-1: and soluble vascular cell adhesion molecule 1.

**Table 2:** Changes in the anthropometric parameters, body composition, and quality of life between baseline and the 6-month follow-up, according to study group, and an analysis of treatment effect.

	Baseline	6 Months	Mean Difference (95% CI)	*p* Value
BMI (kg/m^2^) VLCKD	38.1 ± 1.6	32.8 ± 3.4	−5.3 (−6.9 to −3.6)	<0.001
BMI (kg/m^2^) MedDiet	37.5 ± 2.5	37.3 ± 5.5	−0.1 (−3.8 to 3.5)	0.932
ΔBMI (kg/m^2^)	-	-	−5.2 (−2.0 to −8.3)	0.003
WC (cm) VLCKD	112.4 ± 7.2	93.4 ± 24.8	−19.0 (−36.6 to −1.4)	0.037
WC (cm) MedDiet	111.3 ± 10.1	112.2 ± 10.9	0.9 (−6.4 to 8.2)	0.762
ΔWC (cm)	-	-	−19.9 (−41.0 to 1.1)	0.061
TBF (%) VLCKD	42.4 ± 2.9	35.4 ± 4.8	−7.0 (−10.7 to −3.3)	0.003
TBF (%) MedDiet	42.0 ± 4.1	40.7 ± 3.4	−1.3 (−3.9 to 1.3)	0.242
ΔTBF (%)	-	-	−5.7 (−10.4 to 1.0)	0.022
FFM (%) VLCKD	55.0 ± 5.6	51.9 ± 10.5	−3.1 (−12.0 to 5.8)	0.438
FFM (%) MedDiet	56.1 ± 7.5	58.2 ± 13.5	2.1 (−7.6 to 11.8)	0.584
ΔFFM (%)	-	-	5.2 (−7.0 to 17.4)	0.370
SBP (mmHg) VLCKD	130.1 ± 15.9	125.7 ± 19.9	−4.4 (−14.1 to 5.2)	0.304
SBP (mmHg) MedDiet	134.8 ± 17.3	127.5 ± 21.2	−7.2 (−21.5 to 7.1)	0.205
ΔSBP (mmHg)	-	-	−2.8 (−11.3 to 16.9)	0.662
IWQOL-Lite total score VLCKD	79.0 ± 27.5	37.6 ± 4.3	−41.4 (−75.2 to −7.6)	0.027
IWQOL-Lite total score MedDiet	78.3 ± 35.9	68.5 ± 29.9	−9.8 (−29.3 to 9.8)	0.210
ΔIWQOL-Lite total score	-	-	−31.7 (−66.7 to 3.4)	0.041

Data are mean ± SD. VLCKD: very low calorie ketogenic diet; MedDiet: Mediterranean diet; WC: Waist circumference; TBF: total body fat; FFM: fat-free mass; SBP: systolic blood pressure; IWQOL-Lite: Impact of weight on quality of life-lite.

**Table 3:** Changes in adventitial vasa vasorum density and parameters related to endothelial dysfunction between baseline and the 6-month follow-up, according to treatment group, and an analysis of the treatment effect.

	Baseline	6 Months	Mean Difference (95% CI)	*p* Value
Mean VV VLCKD	1.02 ± 0.2	1.10 ± 0.3	0.1 (−0.1 to 0.2)	0.306
Mean VV MedDiet	1.06 ± 0.2	1.18 ± 0.2	0.1 (−0.1 to 0.2)	0.204
ΔMean VV	-	-	0.0 (−0.3 to 0.2)	0.963
Right VV VLCKD	1.07 ± 0.2	1.12 ± 0.4	0.1 (−0.2 to 0.3)	0.691
Right VV MedDiet	1.15 ± 0.2	1.34 ± 0.3	0.2 (−0.2 to 0.5)	0.207
ΔRight VV	-	-	0.1 (−0.5 to 0.2)	0.461
Left VV VLCKD	1.00 ± 0.3	1.01 ± 0.3	0.1 (0.1 to −0.3)	0.259
Left VV MedDiet	1.06 ± 0.4	1.02 ± 0.4	−0.1 (−0.4 to 0.3)	0.736
ΔLeft VV	-	-	0.2 (−0.5 to −0.2)	0.359
AIP VLCKD	0.1 ± 0.2	−0.0 ± 0.2	0.2 (−0.3 to −0.0)	0.029
AIP MedDiet	0.7 ± 0.2	0.5 ± 0.3	0.2 (−0.4 to 0.7)	0.375
ΔAIP	-	-	0.0 (−0.4 to 0.5)	0.824
TyG index VLCKD	8.8 ± 0.4	8.4 ± 0.5	0.4 (−0.0 to 0.8)	0.060
TyG index MedDiet	9.1 ± 0.3	8.8 ± 0.6	0.3 (−0.7 to 1.4)	0.395
ΔTyG index	-	-	0.1 (−0.7 to 0.8)	0.886
sICAM-1 (ng/mL) VLCKD	389.7 ± 117.9	315.3 ± 74.2	−74.3 (−108.9 to −36.6)	<0.001
sICAM-1 (ng/mL) MedDiet	354.8 ± 146.4	341.4 ± 111.9	−13.3 (−53.9 to 27.1)	0.460
ΔsICAM-1 (ng/mL)	-	-	−60.9 (−115.0 to −6.8)	0.029
sVCAM-1 (ng/mL) VLCKD	1457.9 ± 190.7	1520.0 ± 178.2	62.1 (−41.2 to 165.5)	0.213
sVCAM-1 (ng/mL) MedDiet	1651.0 ± 367.6	1625.0 ± 332.8	−26 (−301.6 to 249.6)	0.818
ΔsVCAM-1 (ng/mL)	-	-	−88.2 (−123.1 to 299.5)	0.390

Data are mean ± SD. VV: vasa vasorum; VLCKD: very low calorie ketogenic diet; MedDiet: Mediterranean diet; AIP: Atherogenic index of plasma; TyG index: Triglyceride-glucose index; sICAM-1: soluble intercellular adhesion molecule 1; sVCAM-1: soluble vascular cell adhesion molecule 1.

**Table 4:** Changes in the newly proposed lipid-based atherogenic scores between baseline and the 6-month follow-up, according to treatment group, together with an analysis of treatment effect.

	Baseline	6 Months	Mean Difference (95% CI)	*p* Value
CRI, VLCKD	4.2 ± 1.0	3.6 ± 0.9	−0.6 (−1.5 to 0.3)	0.161
CRI, MedDiet	4.3 ± 0.4	4.0 ± 0.7	−0.3 (−1.3 to 0.6)	0.329
ΔCRI	-	-	−0.2 (−1.5 to 1.0)	0.437
AI, VLCKD	0.4 ± 0.2	0.2 ± 0.2	−0.2 (−0.5 to 0.0)	0.073
AI, MedDiet	0.7 ± 0.2	0.5 ± 0.3	−0.2 (−0.7 to 0.3)	0.375
ΔAI	-	-	0.0 (−0.4 to 0.3)	0.824
VLDL VLCKD	29.3 ± 12.2	21.5 ± 7.6	−7.8 (−14.6 to 0.9)	0.028
VLDL MedDiet	39.5 ± 12.0	30.4 ± 18.7	−9.1 (−50.7 to 32.0)	0.537
ΔVLDL	-	-	1.2 (−17.4 to 20.0)	0.887

Data are mean ± SD. CRI: coronary risk index; VLCKD: very low calorie ketogenic diet; MedDiet: Mediterranean diet; AI: atherogenic index; VLDL: very low density lipoproteins.

## Data Availability

The data that support the findings of this study are available on request from the corresponding author, [A.L.]. The data is not publicly available as it could compromise the privacy of research participants.
